# Supplementary material for: Neutrophil N1 and N2 Subsets and Their Possible Association with Periodontitis: A Scoping Review
Source: Int J Mol Sci. 2022 Oct 11;23(20):12068. doi: 10.3390/ijms232012068 (PMC9603394; doi:10.3390/ijms232012068)
Supplement: Supplementary file 1 [file ijms-23-12068-s001.zip › Supplementary file S1.pdf]

SUPPLEMENTAL FILE S1 Search strategies

Search Strategies for MEDLINE, EMBASE, WEB OF SCIENCE and LILACS databases were the following:

MEDLINE

((((((((((Neutrophils[MeSH Terms]) OR phenotypes[MeSH Terms]) OR subsets[MeSH Terms]) OR functions[MeSH Terms]) OR Keystone[MeSH Terms]) OR bacteria[MeSH Terms]) OR Oral bacteria[MeSH Terms]) OR Oral Pathogen[MeSH Terms]) OR Periodontal pathogen[MeSH Terms]) OR pathobiont[MeSH Terms]) OR oral microbiota[MeSH Terms])AND ((((((((periodontitis[MeSH Terms]) OR chronic periodontitis[MeSH Terms]) OR experimental periodontitis) OR ligature periodontitis) OR oral gavage periodontitis[MeSH Terms]) OR experimental periodontitis model) OR bone resorption[MeSH Terms]) OR periodontal bone resorption) OR bone destruction[MeSH Terms]) AND ((((Human[MeSH Terms]) OR human samples[MeSH Terms]) OR human periodontium[MeSH Terms]) OR periodontal biopsies[MeSH Terms]).

EMBASE

('periodontitis'OR ‘periodontitis model'/exp OR 't oral gavage'/exp OR ‘ligature method'/exp OR ‘bone resorption'OR ‘bone destruction'OR 'inflammation' OR ‘periodontal bacteria'OR ‘periodontal pathogen'OR ‘oral bacteria' OR ‘oral pathogen' OR ‘keystone pathogen' OR 'pathobiont' OR 'microbiota' OR 'dysbiosis' OR ‘dysbiotic microbiota'/exp OR ‘subgingival microbiota' OR ‘periodontal microbiota' OR ‘bacteria detection'/exp OR ‘bacteria quantification' OR ‘bacteria identification') AND ('neutrophils'/exp OR 'neutrophil'OR ‘neutrophil subsets'OR ‘neutrophil phenotypes'OR 'N1'OR ‘N2'OR ‘Neutrophil characterization'OR ‘Neutrophil AND [embase]/lim

WEB OF SCIENCE

#1 TS=(Periodontitis OR Pathogen OR Experimental periodontitis OR Bacteria OR keystone OR pathobiont)

#2 TS=(neutrophils OR subsets OR differentiation OR phenotypes OR N! OR N2)

#3 #1 AND #2}

LILACS

“PERIODONTITIS" OR “FENOTIPOS DE NEUTRÓFILOS" [Palabras] or “ENFERMEDAD PERIODONTAL" [Palabras] and ("NEUTRÓFILOS") [Palabras]
